# Editorial letter to: Incidence of Ureteric Strictures Following Ureteroscopic Laser Lithotripsy: Holmium: YAG Versus Thulium Fiber Laser

**DOI:** 10.5152/tud.2024.23204

**Published:** 2024-01-01

**Authors:** Mariela Corrales, Aideen Madden, Olivier Traxer

**Affiliations:** 1 Sorbonne University GRC Urolithiasis No. 20 Tenon Hospital, Paris, France; 2 Department of Urology AP-HP, Sorbonne University, Tenon Hospital, Paris, France

Dear Editor,

The original paper by Ahmad Para S et al^1^ analyzed the incidence of ureteral strictures following holmium: yttrium aluminum garnet (Ho:YAG) and thulium fiber laser (TFL) endocorporeal laser lithotripsy (ELL) for ureteral stones. The authors should be commended for their work because this is the first prospective randomized controlled trial comparing the ureteric stricture rates between these 2 laser technologies for ELL. This study concluded that the TFL had a higher incidence of ureteral strictures than the Ho: YAG laser. Our editorial letter aims to clarify and highlight some key points.

In the current paper, the methodology section lacks detail in points that we consider crucial when considering the potential etiology of post-ureteroscopy ureteric strictures. First, the authors neglect to specify the irrigation system used, i.e., by gravity only, or with a manual or automatic pump. The irrigation used while performing ELL is an extremely important point. Prior to clinical evaluations, TFL was expected to result in a higher temperature rise because of its higher water absorption when compared to the traditional Ho:YAG laser.^1^ However, it has been demonstrated that both laser technologies, Ho:YAG and TFL, can induce significant temperature rise during ELL as long as we use high power settings and reduced or absent irrigation.^2,3^ Second, the laser settings are unclear. The authors mention that ELL started with 0.4 J and 8 Hz and was then gradually increased according to surgeon’s preference. Nonetheless, we do not know the maximal total power, nor the total frequency used in each case. The authors^1^ clearly state that both study groups required augmentation in frequency and in energy to achieve the expected stone fragmentation. When working in the ureter, this is an especially dangerous point because it can lead to undesirable results, like ureteral strictures. According to the French Urological Association the maximal laser power admitted in the ureter is 10-15 W.^4^ It has been recently demonstrated in an in vitro study that even if high-power settings result in a greater ablation rate and a reduced operative time, they also cause more thermal damage to the ureter. Moreover, in inexperienced hands, high frequency settings are likely to cause more thermic-related ureteral damage.^3^ It is important to note that a high-power Ho:YAG laser was used in this study (Pulse 100H; Lumenis), indicating that maybe a high frequency was used. Furthermore, nowadays there is no consensus concerning the TFL presets offered by different laser companies. For instance, some companies may go up to 40 Hz for ureteral laser lithotripsy.^5^ A third point relates to the operating surgeons. In terms of preferences for laser settings (personal or presets) for both laser technologies, we do not know whether all ELL cases were performed by the same operator or by different operators. Together, these factors may explain why the TFL caused a higher rate of ureteral strictures. Unfortunately, a complete study outcomes table is missing, which would include the laser settings used for each ureteral stenosis case, the procedure details, and patient-specific factors such as stone and patient characteristics.

Finally, we encourage authors to continue the research in this field due to the lack of randomized control trials mentioning ureteral strictures following ELL with the new TFL. However, prior to reaching any robust conclusions, we would strongly highlight the importance of detailing parameters relating to laser settings and irrigation.
